# Hydroxymethylation and Epigenetic Drugs: New Insights into the Diagnosis and Treatment in Epigenetics of Hepatocellular Carcinoma

**DOI:** 10.1155/2023/5449443

**Published:** 2023-02-09

**Authors:** Wei Ouyang, Ming-Da Wang, Wan-Yin Wang, Chao Li, Lan-Qing Yao, Hong Zhu, Tian Yang

**Affiliations:** ^1^Department of Medical Oncology, The First Affiliated Hospital of Soochow University, Suzhou, Jiangsu, China; ^2^Department of Hepatobiliary Surgery, Eastern Hepatobiliary Surgery Hospital, Navy Medical University (Second Military Medical University), Shanghai, China; ^3^Eastern Hepatobiliary Clinical Research Institute, Third Affiliated Hospital of Navy Medical University, Shanghai, China; ^4^Department of General Surgery, Cancer Center, Division of Hepatobiliary and Pancreatic Surgery, Zhejiang Provincial People's Hospital, Affiliated People's Hospital, Hangzhou Medical College, Hangzhou, Zhejiang, China

## Abstract

Hepatocellular carcinoma (HCC) is a highly lethal and heterogeneous malignancy with multiple genetic alternations and complex signaling pathways. The complexity and multifactorial nature of HCC pose a tremendous challenge regarding its diagnosis and treatment. Emerging evidence has indicated an important regulatory role of epigenetic modifications in HCC initiation and progression. Epigenetic modifications are stably heritable gene expression traits caused by changing the accessibility of chromatin structure and genetic activity without alteration in the DNA sequence and have been gradually recognized as a hallmark of cancer. In addition, accumulating data suggest a potential value of altered hydroxymethylation in epigenetic modifications and therapeutics targeting the epigenetically mediated regulation. As such, probing the epigenetic field in the era of precision oncology is a valid avenue for promoting the accuracy of early diagnosis and improving the oncological prognosis of HCC patients. This review focuses on the diagnostic performance and clinical utility of 5-hydroxymethylated cytosine, the primary intermediate product of the demethylation process, for early HCC diagnosis and discusses the promising applications of epigenetic-based therapeutic regimens for HCC.

## 1. Introduction

Hepatocellular carcinoma (HCC) represents the most frequent type of primary liver cancer and accounts for more than 90% of all liver cancer cases worldwide, with an annually increasing incidence and a dismal long-term prognosis [1, 2]. Epigenetic modifications contribute to the complexity and multifactorial nature of HCC as a significant mechanism, and molecular genetic alterations that affect epigenetic modification were reported to be critical factors in HCC carcinogenesis during the preneoplastic stage [3, 4]. The epigenetic regulation of chromatin consists of DNA methylation, nucleosome histone variants, post-translational histone modifications (PTMs), and noncoding RNAs [5]. Of these, DNA methylation is one of the most predominant research hotspots in epigenetics to date. Meanwhile, aberrant methylation processes and intermediates are also suggested to be essential hallmarks of HCC, with a great potential promise for early HCC diagnosis and therapeutic guidance [6].

Despite improvements in the early diagnosis and treatment of HCC, novel biomarkers for earlier diagnosis and better therapeutic interventions are urgently needed to improve long-term outcomes. 5-Hydroxymethylcytosine (5hmC), an intermediate product of the demethylation of 5-methylcytosine (5 mC) by ten-eleven translocation proteins, serves as an eminent epigenetic modification of DNA in the mammalian cells [7, 8]. It is also known as the “sixth base” of DNA [9, 10] and plays an essential role in gene regulation, cell development, and tumorigenesis [11–16]. Facing and considering the unsatisfactory diagnostic accuracy of the traditional serum biomarkers in the early diagnosis of HCC and the suboptimal effectiveness of current systemic therapies for patients with advanced disease, it is of utmost importance to identify novel potential biomarkers and effective therapeutic strategies for different stage HCC [17, 18]. Recent advances in the high-throughput sequencing technologies (e.g., nano-hmC-Seal [19] and hMe-Seal [20]) have made it possible to uncover the genome-wide 5hmC profiling of hematological or solid tumors. The specific genomic distribution pattern of 5hmC revealed that this mark was highly enriched at promoters and enhancers of transcriptionally active genes [21]. An increasing amount of studies have indicated that the level of 5hmC in various solid tumors decreased significantly compared to adjacent tissues [20, 22–31], suggesting an essential role of 5hmC in tumorigenesis and progression as well as its potential utility in tumor diagnosis. At the same time, there is an immense promise for exploring novel epigenetic biomarkers for cancer due to the limitations of the current traditional histopathology-based approaches for HCC detection in clinical practice. Moreover, given that epigenetic modifications, for example, reversible enzymatic reactions and specific protein-protein interactions (e.g., DNA methylation and PTM processes) are highly flexible and more susceptible to pharmacological interference, such novel strategies may pave new promising avenues toward therapeutic HCC [32].

Herein, we summarize the most recent progress in the diagnostic applications of 5hmC in HCC and evaluate its latent value of being a promising diagnostic biomarker for HCC, highlighting the emerging strategies of epigenetics-based targeted drugs in the era of HCC treatment.

## 2. 5hmC Serves as a Promising Early Diagnostic Biomarker in HCC

**Figure 1:**
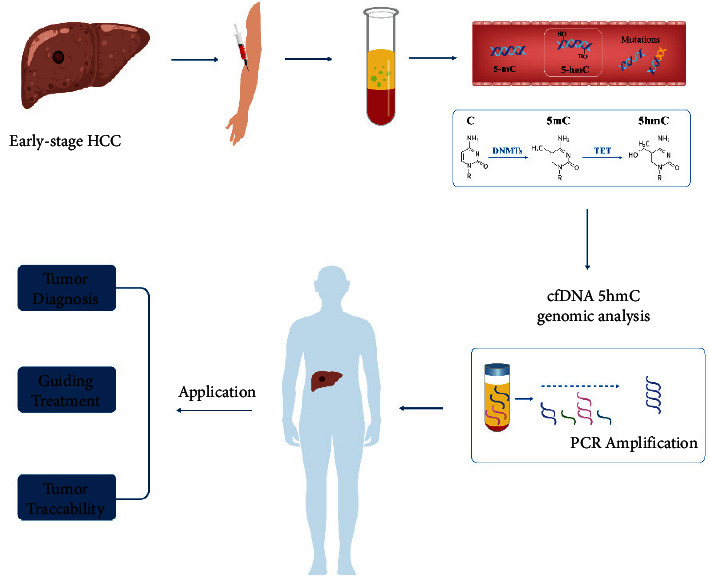
Circulating 5hmC as a novel biomarker of epigenetic modification for the early diagnosis of HCC. Small fragments of tumor DNA exist from the peripheral blood with an early-stage HCC, containing marks such as 5mC and 5hmC that are associated with epigenetic alterations in tumors. 5hmC as stable epigenetic marks associated with gene regulation and tumorigenesis, which is a staple of the demethylation process. Sequencing analysis of 5hmC revealed that 5hmC is markedly enriched in tissue-specific and tumor-specific differentially methylated regions, and discriminates between HCC and non-HCC patients with higher sensitivity and specificity versus traditional tumor biomarkers, suggesting a new strategy for the early diagnosis of tumors including HCC as a novel liquid biopsy modality. HCC, hepatocellular carcinoma; 5hmC, 5-hydroxymethylated cytosine; 5mC, 5-methylcytosine; C, cytosine; DNMT, DNA methyltransferases; TET, ten-eleven translocation.

Paralleling the remaining solid tumors, previous studies have demonstrated that the 5hmC level was significantly decreased in an advanced cirrhosis and early HCC stage and was closely associated with poor prognosis and tumor progression [33, 34]. As such, 5hmC appears to be an impressive biomarker for early diagnosis and prognostic prediction of HCC (The flow diagram of 5hmC for HCC detection is shown in Figure 1). Several studies have proved the good diagnostic accuracy of 5hmC for detecting HCC, suggesting a potential prospect of clinical application. A previous study by Chen et al. [35] utilized a constructed mass spectrometer technique to examine 5hmC levels in HCC and revealed the possibility of 5hmC as a biomarker for early detection and prognosis of HCC. Cai et al. [29] established a 32-genes-based 5hmC diagnostic model using circulating cell-free DNA and exhibited a great performance for distinguishing early-stage HCC from non-HCC (training set: area under curve (AUC) = 0.92, 95% confidence interval (CI): 0.91–0.94; validation set: AUC = 0.88, 95%CI: 0.86–0.91), which appeared to outperform *α*-fetoprotein (AFP) when detecting an early HCC and may compensate for the plight of those patients with early HCC misdiagnosed due to AFP. Additionally, this model could be used to distinguish the patients with early small tumors (≤2 cm) accurately from high-risk patients with chronic liver disease (validation set: AUC = 0.85, 95%CI: 0.81–0.89), confirming the clinical application potential of 5hmC for the early detection of HCC. Another noninvasive diagnostic approach based on 5hmC signatures of plasma cell-free DNA effectively distinguished patients with HCC from cirrhotic patients and healthy controls with a relatively high AUC of 0.93 [36]. Song et al. [20] constructed a diagnostic model using the cell-free 5hmC signature with success in distinguishing HCC patients from hepatitis B virus infection and healthy controls, as well as monitoring treatment outcome and disease recurrence. Meanwhile, the distinct features of cell-free 5hmC yielded accurate predictions for specific cancer types and tumor stages. Given the limited number of studies regarding the mechanisms by which 5hmC regulates the pathogenesis of HCC [32], further basic research is still needed on HCC-related studies caused by 5hmC. Nevertheless, the current robust results about clinical applications of 5hmC as a molecular biomarker to guide the diagnosis of HCC and even for monitoring prognosis and recurrence are promising and advantageous compared to traditional biomarkers. The Cell-free 5hmC provides a novel dimension of informativeness for liquid biopsy-based diagnosis and surveillance.

## 3. Epidrugs: Targeting Epigenetic Marks in HCC Therapies

Intricate biological processes derived from aberrant gene regulation and epigenetic mutations have participated in developing HCC. It is well established that telomerase reverse transcriptase (TERT), Catenin *β*1 (CTNNB1), and TP53 are the most commonly mutated genes in association with the HCC development, yet the exploration of targeted therapies against these oncogenic driver genes genetic drivers remains unsuccessful [37, 38], highlighting the importance of developing new targeted therapeutics for patients with HCC. Following the evolution of high-throughput sequencing technologies and the accumulation of knowledge in the field of epigenetics, mutations in epigenetically modified genes have been indicated to be closely correlated with the development and progression of HCC, with up to 50% of tumors harboring relevant mutations [39]. Given this, research on epigenetic drugs (epidrugs) has received much interest and extensive attention in clinical practice [40].

**Figure 2:**
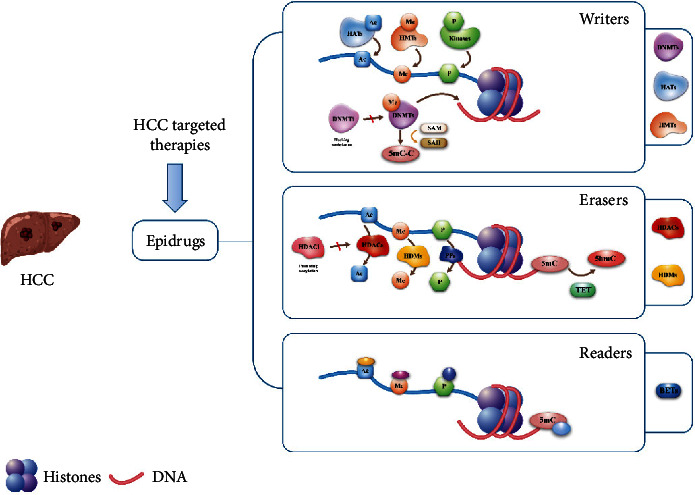
Schematic diagram: the regulation mechanism of DNA epigenetic modifiers on gene expression and epigenetic drugs. Epigenetic modifiers include epigenetic writers, readers, and erasers. HCC, hepatocellular carcinoma; 5hmC, 5-hydroxymethylated cytosine; 5mC, 5-methylcytosine; Epidrugs, epigenetic drugs; DNMTs, DNA methyltransferases; HATs, histone acetyltransferases; HMTs, histone methyltransferases; HDMs, histone demethylases; HDACs, histone deacetylases; BETs, Bromo- and extra-terminal; DNMTi, DNMT inhibitors; HDACi, HDAC inhibitors.

Epidrugs are well-characterized small molecule inhibitors that mainly target epigenetic genes or enzymes and are divided into three categories: writers, readers, and erasers [32, 41]. Writers are enzymes that add covalent modifications to DNA and histones. They include DNA methyltransferases (DNMTs), which transfer methyl groups from S-adenosyl methionine (SAM) to cytosine bases of CpG dinucleotides at gene promoters and regulatory regions [42]. Histones are methylated on lysine and arginine residues to develop complex PTM. Catalytic enzymes in histone methylation involve histone methyltransferases (HMTs) and histone acetyltransferases (HATs), which exert a crucial impact on chromatin remodeling and gene expression [32]. Shanmugam et al. [43] further illustrated the link between aberrant epigenetic histone modifications and carcinogenesis and assessed their possible impacts on clinical outcomes of patients with HCC. Erasers (e.g., histone demethylases (HDMs) or histone deacetylases (HDACs), however, regulate DNA demethylation to reverse writers' functions. Moreover, epigenetic modifications are recognized by the third group of proteins named readers, the unique structural domains endowed with specific covalent modifications that function as effector proteins (e.g., methyl-binding domain proteins or Bromo- and extra-terminal (BETs) domain proteins) (Figure 2). The modification process described above emphasizes the complexity and reciprocal interaction of epigenetic regulatory mechanisms that underline the promising epidrugs.

### 3.1. DNA Methylation Inhibitors

With epidrugs being recognized as a promising targeted therapeutic approach for treating and reversing cancer drug resistance, particularly notable for therapies with DNA methylation inhibitors and histone acetylation inhibitors, the following section emphasizes the previous methods. The current epidrugs have mainly been applied in hematological malignancies and exerted an anti-tumor effect via the inhibition of DNMTs [44] and HDACs [45], while seldom used to treat solid tumors due to the high rates of acquired drug resistance and lack of specific therapeutic targets. Either the first-generation DNMT inhibitors (DNMTi) (e.g., azacitidine [46] and Decitabine) or the second-generation DNMTi (e.g., guadecitabine (SGI-110)) developed to improve stability and overcome short-halflives, and HDAC inhibitors (HDACi) (e.g., vorinostat and panobinostat) appear to be widely applied in hematological tumors with the U.S. Food and Drug Administration approved. Additionally, the combination of other drugs in solid tumors has yielded greater anticancer effects than that induced by either drug alone [47], although there remain several mild adverse effects [48]. Emerging evidence suggests that DNMTi can be successfully applied in managing HCCs. Liu et al. [49] demonstrated that DNMTi significantly inhibited the colony formation of sorafenib-resistant HCC cells, indicating a therapeutic effect on resistant HCCs to sorafenib. Mei et al. [50] and Fan et al. [51] revealed that low-dose decitabine was effective in resensitizing resistant HCC cells to sorafenib alone or in combination with chemotherapy or immunotherapy in treating advanced HCC. Similarly, second-generation DNMTi is equally effective as low-dose guadecitabine alone or combined with oxaliplatin [52] or sorafenib [53], Gailhouste et al. [46] Also, DNMTi has therapeutic implications for HCC by promoting the reactivation of aberrantly silenced tumor suppressor genes, thereby enhancing sensitivity to sorafenib in HCC cells. As for combined therapies, DNMTi improved the efficacy of treatments such as chemotherapy, and equally, the combined immunotherapy modality holds advantages in treating HCC, benefiting from immunotherapy and improving outcomes [54]. Recently, a phase Ib clinical trial (NCT03257761) was conducted to evaluate the efficacy of guadecitabine in combination with durvalumab for treating gastrointestinal tumors, including HCC, and suggested the potential benefit of the combined therapy in selected patients. Furthermore, CM-272, a novel targeted dual-acting small molecule inhibitor of HMTs and DNMTs, exhibited potent anti-tumor activity against HCC cell lines by synergistically downregulating the expressions of DNMT1 and G9a [55], yet further clinical trials are needed to demonstrate its effectiveness and safety.

The above study found that low doses of DNMTi were effective in reducing the incidence of drug-related toxic effects, but common adverse events observed in the study were neutropenia, thrombocytopenia, anemia, nausea, and fatigue. Besides, neurological toxicity has been reported in a nonsmall cell lung cancer study with decitabine in combination with valproic acid [56].

### 3.2. HDAC Inhibitors

As aberrant histone deacetylation causes silencing of tumor suppressors in many of the known cancers, and research has shown aberrant expression of HDAC in HCC [57, 58], thus HDACi offers a promising approach to treat HCC. The pan-HDACi panobinostat [59] and pan-HDACi belinostat [60] have been proved to be effective treatment strategies for HCC by inhibiting the proliferative effect of HCC [61]. Other epigenetic therapies, such as Trichostatin (TSA) [62, 63] and Reminostat, are currently approved for clinical use and exhibit excellent anti-tumor effects in the HCC treatment [64]. Recent evidence demonstrated that targeting epigenetic modification strategies is capable of enhancing immune recognition of tumor cells hence the combinations of immunotherapy yield synergistic effects and induce robust anti-tumor responses [54, 65]. Immuno-combination therapies are a rapidly expanding field in targeting anti-tumor therapies, including HCC. The combinations of pan-HDACi belinostat with anti-CTLA-4 and anti-PD-1 antibodies have been studied to improve the anti-tumor efficacy of immune checkpoint inhibitors in a murine HCC model [66]. Correspondingly a multitude of clinical trials of epidrugs in conjunction with immune checkpoint inhibitors by HDACi are ongoing and are expected to yield implications for the clinical practice of immune conjugation strategies against HCC. Besides, therapeutic strategies including HDACi combined with other analogs also have been suggested to have anti-HCC potential and warrant further validation (A summary of the clinical stages of the different epidrugs in the treatment of HCC is presented in Table 1).

Regardless of the positive anti-tumor efficacy of HDACi against HCC, adverse side effects associated with HDACi deserve to be taken into account, notably when HDACi is used in combination with a variety of therapeutic drugs. Hepatic impairment mainly due to cumulative dose toxicity of the drug has been observed in clinical trials, including hyperbilirubinemia, elevated liver enzymes, and other dominant toxicities comprising fatigue, abdominal pain, anemia, and vomiting.

## 4. Conclusion

Aberrant epigenetic alterations are implicated in the pathogenesis of HCC. Epigenetic modifications include DNA methylation, hydroxymethylation, histone modifications, which can exert the differential expression of the genome and chromatin at the cellular transcriptome level. The development of high-throughput sequencing technologies has revealed a genome-wide map of 5hmC and low levels of 5hmC in the context of an early-stage HCC and associated with HCC progression, exploring it as a biomarker to serve in the field of diagnosis. Identification of 5hmC levels by liquid biopsy improves the diagnostic accuracy of HCC, making it possible to detect HCC earlier in large high-risk populations. Furthermore, with the growing establishment of epigenetic markers for the diagnosis and prognosis of solid tumors, epigenomic-targeted therapies may provide more combination strategies for treating HCC in the near future. Particularly, DNMTi and HDACi have been well-tested alone or in combination with other categories of drugs for treating HCC. Subsequent exploration of epigenetic modifications, including abnormal DNA methylations and histone modifications, is warranted to ascertain potential biomarkers for HCC diagnosis and formulate effective combined treatment strategies on the basis of epigenetic modification inhibitors in an attempt to overcome adverse effects and improve anti-tumor efficacy with better pharmacodynamics. However, it is notable that the lack of approved epidrugs available in the domain of HCC to date, hopefully the solutions offered to address the limitations referred therein will yield the optimal results in future. In conclusion, it is worthwhile to work towards a better comprehension of the mechanisms of epigenetic modifications and the interactions of epigenetic modifiers as a means to develop other more effective biomarkers and epigenomic therapies in the clinical setting.

## Figures and Tables

**Table 1:** List of the various clinical phases of epigenetic drugs in the treatment of HCC.

Epigenetic drugs	Preclinical stage	Clinical trials	Ref./clinical trial number	Clinical trial start
Mechanism of action				
DNMT inhibitors				
Azacitidine	*∗*		[46]	
Decitabine		*∗*	Phase I/II (NCT01799083)	Dec 2012
CM-272	*∗*		[55]	
Guadecitabine (SGI-110)		*∗*	Phase II (NCT01752933)	Dec 2012
			Phase I (NCT03257761)	Feb 2018
HDAC inhibitors				
Vorinostat		*∗*	Phase I (NCT01075113)	Aug 2010
Belinostat		*∗*	Phase I/II (NCT00321594)	May 2006
Panobinostat		*∗*	Phase I (NCT00873002)	Mar 2009
			Phase 1 (NCT00823290)	Jan 2009
Trichostatin	*∗*		[61, 62]	
Reminostat		*∗*	Phase II (NCT00943449)	Jul 2009
			Phase I/II (NCT02400788)	Apr 2013

Abbreviations; DNMT : DNA methyltransferases; HDAC: histone deacetylases.

## Data Availability

The data supporting this mini-review are from previously reported studies and datasets, which have been cited. The processed data are available from the corresponding author.
